# New Stable Cell Lines Derived from the Proximal and Distal Intestine of Rainbow Trout (*Oncorhynchus mykiss*) Retain Several Properties Observed In Vivo

**DOI:** 10.3390/cells10061555

**Published:** 2021-06-19

**Authors:** Rolando Pasquariello, Nicole Verdile, Radmila Pavlovic, Sara Panseri, Kristin Schirmer, Tiziana A. L. Brevini, Fulvio Gandolfi

**Affiliations:** 1 Department of Agricultural and Environmental Sciences, University of Milan, 20133 Milano, Italy; rolando.pasquariello@unimi.it (R.P.); nicole.verdile@unimi.it (N.V.); 2 Department of Health, Animal Science and Food Safety, University of Milan, 20133 Milano, Italy; radmila.pavlovic1@unimi.it (R.P.); sara.panseri@unimi.it (S.P.); tiziana.brevini@unimi.it (T.A.L.B.); 3 Department of Environmental Toxicology, Eawag, Swiss Federal Institute of Aquatic Science and Technology, 8600 Dübendorf, Switzerland; kristin.schirmer@eawag.ch; 4 Department of Environmental Systems Science, ETH Zürich, 8092 Zürich, Switzerland; 5 School of Architecture, Civil and Environmental Engineering, EPFL Lausanne, 1015 Lausanne, Switzerland

**Keywords:** fish, intestinal cell lines, intestinal stem cells, enterocyte differentiation markers, intestinal barrier in vitro, cell differentiation, nutrition

## Abstract

We derived two novel cell lines from rainbow trout (RT) proximal (RTpi-MI) and distal intestine (RTdi-MI) and compared them with the previously established continuous cell line RTgutGC. Intestinal stem cells, differentiating and differentiated epithelial cells, and connective cells were found in all cell lines. The cell lines formed a polarized barrier, which was not permeable to large molecules and absorbed proline and glucose. High seeding density induced their differentiation into more mature phenotypes, as indicated by the downregulation of intestinal stem cell-related genes (i.e., *sox9*, *hopx* and *lgr5*), whereas alkaline phosphatase activity was upregulated. Other enterocyte markers (i.e., sglt1 and pept1), however, were not regulated as expected. In all cell lines, the presence of a mixed population of epithelial and stromal cells was characterized for the first time. The expression by the stromal component of *lgr5*, a stem cell niche regulatory molecule, may explain why these lines proliferate stably in vitro. Although most parameters were conserved among the three cell lines, some significant differences were observed, suggesting that characteristics typical of each tract are partly conserved in vitro as well.

## 1. Introduction

Consumption of carnivorous fish and crustacean such as salmon, trout, sea bass, sea bream and shrimp, is constantly growing; to satisfy the consumers’ demand, these species are increasingly farmed in intensive systems [1]. Until recently, fishmeal and fish oil, obtained by processing small oily fish species caught for non-food purposes by so-called industrial fisheries, have extensively been used as major sources of protein and energy in fish feeds [2]. This constitutes a severe limitation to the sustainability of the aquaculture industry, which is gradually substituting such declining natural resources with alternative feeds [3]. However, the ability of alternative diets to replace increasing amounts of their fish-based counterparts in carnivorous fish species has been limited by adverse responses, often species-specific, that depend on the nutritional- and anti-nutritional characteristics and dietary inclusion levels of different ingredients. This difficulty not only impairs feed efficiency, but also compromises animal welfare, causing severe gastrointestinal pathologies that include diarrhea [4], decreased nutrient absorption [5], intracellular lipid accumulation [6], gastric bloating [7], intestinal inflammation [8], and cancer [9,10]. The current lack of mechanistic knowledge of the cellular and molecular mechanisms that determine the adverse reactions of the fish intestinal epithelium limits our capacity to support the development of effective alternative diets. In this perspective, the development of an advanced in vitro platform would be a useful tool for screening new feed formulations before performing in vivo feeding trials, substantially reducing time, costs, and the number of experimental animals, as recently proposed [11].

Current intestinal in vitro models are most advanced for mammalian systems and are based either on immortalized cell lines or on organoids. The latter are 3D structures that originate from intestinal stem cells grown inside collagenous matrices [12,13]. Organoids, in this case also called enteroids, recreate structures resembling both the crypt and the villus compartment of the intestinal epithelium [14]. They provide excellent models for studying the development and the pathology of the intestine. However, their closed design with the intestinal epithelium facing the inner surface of a cystic structures embedded in a gelatin makes them unsuitable for studying the digestive and adsorption mechanisms. For this reason, cell lines are used for studying the interactions between intestinal cells and a variety of physiological, toxicological, and pathological challenges because their apical surface can be easily exposed to the substances under study, they can be propagated vigorously, and easily standardized [15]. The downside is the limited variety of cell types and, often, their poor differentiation [16].

However, when working on farmed fish, the choice of immortal intestinal cell lines is limited to rainbow trout (*Oncorhynchus mykiss*). The RTgutGC [17] and the RTgutF [18] cell lines have been derived from this species, and at present are the only available stable intestinal cell lines, being of epithelial and fibroblast nature, respectively. Rainbow trout is also the only aquaculture species where intestinal stem cells have been characterized in detail [19].

We have recently described that the cell types of the epithelium lining the rainbow trout proximal intestine differ from those of the distal portion and that the two regions are characterized by different renewal rates [20]. With the perspective of developing an in vitro platform that mimics the intestinal mucosa, the aim of this work was to derive new stable rainbow trout intestinal cell lines from both the proximal and the distal intestinal regions. We investigated their ability to maintain some of the differences that are present in vivo, and the presence of a heterogeneous cell population. We also assessed the ability of the newly derived cell lines to form a functional epithelial barrier by means of discriminating between small and large molecules and to respond to a mild differentiation stimulus. Finally, their properties were compared with those of the RTgutGC cell line to assess possible functional differences.

## 2. Materials and Methods

Unless otherwise stated, all reagents were purchased from Sigma-Aldrich, Milan, Italy.

### 2.1. Derivation of New Intestinal Cell Lines

Tissue explants were isolated from a total of 10 rainbow trout (*Oncorhynchus mykiss*), of up 500 g in weight, which were kindly donated from fish culture ponds at Laghi Verdi s.n.c. trout farm (Como, Lombardia, Italy). Euthanasia was performed according to Annex IV of the EU guideline 2010/63. After dissection, the intestine was isolated and quickly rinsed with Dulbecco’s phosphate-buffered saline (PBS, cat. No. D5652) supplemented with 10,000 units/mL penicillin, 10.0 mg/mL streptomycin, and 25.0 μg/mL amphotericin B (antibiotics, cat. No. A5955). The proximal intestine was sampled immediately downstream of the pyloric caeca and the distal intestine was sectioned caudally of the ileum-rectal valve. Samples from the two regions were processed separately but using the same protocols. The intestinal tracts were cut longitudinally, and the lumen was rinsed with PBS to eliminate the mucous layer lining the mucosa. Thereafter, each portion of the intestinal wall was cut into approximately 20–30 fragments of about 1 mm^2^ that were rinsed 3 times using PBS, and further 2 times using what we define as L-15 complete medium, made of Leibovitz’s L-15 (Thermo-Fisher Scientific, Waltham, MA, USA cat n. 21083027), supplemented with 5% fetal bovine serum (FBS, Sigma cat. n. 10270106, Gibco, Waltham, MA, USA), and antibiotics, as indicated above. On average, 5 tissue fragments derived from each tract of each animal were carefully placed in 25 cm^2^ culture flasks (Sarstedt, Nümbrecht, Germany) with the epithelial surface facing downwards towards the surface previously coated with 0.1% gelatin derived from pig skin (cat n. G1890). Finally, the volume of L-15 medium was gradually adjusted to cover all the tissue fragments. Culturing of the tissue fragments was performed at 20 °C in an incubator under ambient atmosphere. L-15 complete medium was replaced once a week. To propagate cells further, tissue fragments were mechanically removed, and cells in each flask were detached using Trypsin-EDTA (cat. n. T3924). Cell suspensions obtained from the same tract of the same fish were pooled into a new T25 flask.

### 2.2. Cell Culture

We compared the two newly established cell lines with the RTgutGC cells that had previously been derived from the distal segment of a rainbow trout intestine [17]. Experiments with the new cell lines were performed between passage 8 and 25, whereas RTgutGC cells were transferred from the laboratory of K. Schirmer at passage 73 and experiments were performed between passages 80 and 100.

For more than 18 months, cells were passaged regularly at a 1:3 ratio every 5–7 days after having reached 90–95% confluency in L-15 complete medium. RTpi-MI and RTdi-MI cells were stored in liquid nitrogen, as previously described [17], and survived repeated freezing/thawing cycles.

At each passage, cells were detached using Trypsin-EDTA solution (cat. No. T3924-100 ML) for 1 min, followed by blocking the enzyme with 5% FBS.

To evaluate proliferation, cells were seeded in 4-well plates at 37,000 cells/cm^2^, equal to 40–50% confluency (Nunc, cat n. 176740, Thermofisher Scientific, Waltham, MA, USA). From day one to six, cells of 3 wells were detached daily and counted using a cell count chamber. Cell doubling time of each cell line was calculated as previously described [21].

To study the effect of seeding density on growth and differentiation, cell lines were seeded in 4-well plates at 100,000 (confluent, which will be indicated as low seeding density) or 200,000 (superconfluent, which will be referred as high seeding density) cells/cm^2^ as previously described [17] and cultured for 7 days.

### 2.3. Expression of a Gene Panel for Cell Type Characterization

At 90–95% confluency, cells were detached using Trypsin-EDTA solution, as described above; the cell suspension was centrifuged at 800× *g* for 5 min, pellets were washed twice in PBS, snap-frozen in liquid nitrogen, and stored at −80 °C until RNA extraction and gene expression analysis.

We analyzed the expression of the following genes, representative of different cell sub populations: (i) SRY-box 9 (*sox9*) and leucine-rich repeat-containing G-protein-coupled receptor 5 (*lgr5*), which are intestinal stem cell and stem cell niche markers, respectively [13]; (ii) homeodomain-only protein (*hopx*), a marker of partially differentiated, transient amplifying cells [13]; (iii) Zonula occludens 1 (*zo-1*), Claudin-3 (*clnd3*), and E-cadherin (*E-cad*), as epithelial cell markers; (iv) Type I Collagen (*col1a1*) and Vimentin (*vim*) as connective tissue markers; (v) intestinal alkaline phosphatase (*iap*), peptide transporter 1 (*pept1*), sodium–glucose/galactose transporter 1 (*sglt1*), and fatty-acid-binding protein 2 (*fabp2*) as markers of mature enterocytes; and mucin 1 (*muc1*), a goblet cell marker.

Poly(A)mRNA samples were obtained from 3 samples of each cell line using a Dynabeads mRNA DIRECT micro-kit (Deutsche Dynal, Hamburg, Germany) following the manufacturer’s instructions. Samples were converted to complementary DNA (cDNA) using an iScript Advanced cDNA Synthesis Kit for RT-qPCR (Bio-Rad, Hercules, CA, USA). Briefly, each sample was brought to 15 µL using nuclease-free water; then, 4 µL 5X iScript Advanced Reaction Mix and 1 µL iScript Advanced Reverse Transcriptase were added to each RNA sample. Reverse transcription (RT) was performed by incubating the samples for 20 min at 46 °C and inactivating the reaction for 1 min at 95 °C using a DNA Thermal Cycler (Perkin Elmer). The cDNA samples were stored at −20 °C until PCR amplification. Qualitative PCR was performed using GoTaq^®^ G2 Flexi DNA Polymerase kit (Promega Corporation, Madison, WI, USA). Each PCR reaction was run to 25 µL using 10.8 µL RNase-free water, 5 µL 5X Green GoTaq Flexi Buffer, 1.5 µL 25 mM MgCl, 0.5 µL 10 mM dNTPs, 5 µL 10 mM primer mix, 0.2 µL GoTaq G2 Flexi DNA polymerase, and 2 µL cDNA sample. The PCR program was: 95 °C for 2 min for the first cycle of denaturation, followed by 40 cycles of 95 °C for 30 s (denaturation), 60 °C for 30 s (amplification) and 72 °C for 5 min (extension), and final extension at 72 °C for 5 min. For each PCR reaction, a negative control was run to exclude contaminations. PCR products were analyzed using electrophoresis on a 2% agarose gel that was stained using ethidium bromide (ThermoFisher Scientific, Waltham, MA, USA). A molecular marker of 50 base pair (bp) gaps (ThermoFisher Scientific, Waltham, MA, USA) was run for each gel to confirm the length of the PCR fragments. The primers used are listed in Table S1. For all the primers, sequencing of the PCR products was performed by Eurofins Genomics (Ebersberg, Germany, Europe). Sequence validations were obtained by alignment against the rainbow trout transcriptome.

### 2.4. Immunofluorescence

For zonula occludens-1 (Zo-1) and collagen type I staining (Col1a1), cells were fixed in 4% paraformaldehyde for 30 min at room temperature. For Zo-1 staining, cells were incubated overnight at 4 °C with an FITC conjugated anti-Zo-1 antibody (1:100 dilution, cat. No. 339188, Life Technologies). Aspecific bindings were prevented by incubating cells in 5% bovine serum albumin and 0.3% Triton X-100 in PBS for 30 min at room temperature. For collagen type I staining, cells were permeabilized with 0.2% Triton X-100 in PBS for 15 min at room temperature. Aspecific bindings were prevented by incubating cells in 3% bovine serum albumin and 0.5% Triton X-100 in PBS for 30 min at room temperature. Afterwards, cells were stained using an anti-collagen type I antibody (1:40 dilution, cat. No. ABIN237021, antibodies-online GmbH, Aachen, Germany) after incubating overnight at 4 °C. Subsequently, cells were incubated with secondary antibody Alexa Fluor^TM^ 488 goat anti-rabbit (1:1000, Life Technologies Corporation, A27034, Carlsbad, OG, USA) for 1 h at room temperature. For Sglt1, Pept1 and Fabp2 immunolabelling, cells were seeded at low and high density, as described above, and directly fixed at day 7 after culture. Then, aspecific bindings were prevented by incubating cells in 10% goat serum (cat. No. G9023) for Sglt1 and Pept1 or 10% donkey serum (cat. No. D9663) for Fabp2; all the sera were diluted in PBS and incubation for 30 min at room temperature was performed. Samples were then incubated with anti-PepT1 mouse monoclonal antibody (Santa Cruz Biotechnology, sc-373742, Heidelberg, Germany) 1:100 diluted in PBS, anti-Sglt1 rabbit polyclonal antibody (Millipore Corporation, 07-1417, Darmstadt, Germany) 1:100 diluted in PBS, or anti-FABP2 goat polyclonal antibody (Novus Biologicals, Minneapolis, MN, USA) 1:150 diluted in PBS, for 60 min at room temperature. Primary antibody specificity was previously validated in rainbow trout intestines [19]. Subsequently, cells were incubated with appropriate secondary antibody Alexa Fluor^TM^ 594 goat anti-mouse (Life Technologies Corporation, cat. No. A11058, Willow Creek Road, OG, USA) or with Alexa Fluor^TM^ 594 goat anti-rabbit (Life Technologies Corporation, cat. No. A11012, Carlsbad, OG, USA) or Alexa FluorTM 594 donkey anti-goat (Life Technologies Corporation, A11058 Willow Creek Road, OG, USA), all diluted 1:250 in PBS for 30 min at room temperature. Nuclei were always counterstained with 4′,6-diamidino-2-phenylindole (DAPI) for 20 min at room temperature. Images were acquired using an Eclipse TE200 microscope (Nikon, Tokyo, Japan).

### 2.5. Epithelial Barrier Analysis and Permeability of Large and Small Molecules

All cell lines were seeded at a density of 57,000/cm^2^ onto a permeable polyethylene (PET) membrane insert (Greiner BioOne, ThinCert cat. No. 665640, 0.4 μm pore size, 1.13 cm^2^ surface growth area) in order to form a cell monolayer, as previously performed [18,22], and maintained at 20 °C.

Transepithelial electrical resistance (TEER) was measured over the course of 21 days of culture using an EVOM2 Epithelial Voltohmmeter with STX2 electrode (World Precision Instruments, Berlin, Germany) as described by Geppert et al. [23]. TEER was calculated by subtracting the values without cells from the values with cells. TEER values are given as Ohms × cm^2^.

For measuring paracellular permeability and absorption, 600 µL of L-15 complete medium supplemented with a mixture of 100 µg/mL 4 KDa FITC-Dextran (Sigma Aldrich; cat.No. FD4-250MG), 918 µg/mL D-Glucose-6,6-d2 (D2-Glucose, cat.no 282650) and 75 µg/mL L-Proline-2,5,5-d3 (D3-proline, cat. No. 791261) was pipetted in the apical compartment of the transwell inserts, and 1.2 mL PBS was pipetted into the basolateral compartment, as recommended by the manufacturer.

FITC-Dextran was quantified by high-performance liquid chromatography coupled to fluorometric detection (HPLC-FLD), whereas D2-glucose and D3-proline were quantified using high-performance liquid chromatography coupled to a high-resolution mass spectrometer q-Exactive Orbitrap (HPLC-Orbitrap-HRMS). Detailed operative conditions are given in the Supplementary Materials (see the permeability test). Both analytical methods were fully validated for linearity (R > 0.1), accuracy (Coefficient of Variance, CV, in the range 8.9–12.1), recovery (>89.0%) and limit of quantification (FITC-Dextran = 0.5 µg/mL; Glucose D2 = 5 µg/mL; Proline D3 = 5 µg/mL). As a preliminary characterization, we determined that it takes 24 h for all 3 markers to reach the concentration equilibrium between the apical and basolateral compartments on the PET membrane without cells. During these preliminary experiments, we also found that 20% of the starting concentration of all 3 markers was absorbed by the membrane (data not shown). Therefore, absolute quantification of all molecules was performed after collecting 100 µL samples from both the apical and the basolateral compartments at 24 h to observe the paracellular and transcellular flux. Deuterated pyruvate was measured to assess if D2-glucose was metabolized.

### 2.6. Fluorescent In Situ Hybridization

Due to the lack of commercially available fish-specific antibodies, the identification of cells expressing genes specific for intestinal stem cells (*sox9* and *lgr5*) and transient amplifying cells (*hopx*) were visualized using fluorescent in situ hybridization (FISH) using Multiplex Fluorescent Reagent Kit V2 (RNAscope technology, Advanced Cell Diagnostics, San Francisco, CA, USA) according to the manufacturer’s instructions. Probes were designed by Advanced Cell Diagnostics (ACD) using the rainbow trout sequences whose primers are listed in Table S1. The peptidyl-prolyl cis-trans isomerase B (*ppib*) gene was used as a specific marker of both mature and proliferating epithelial cells [19]. Cells were fixed in 10% neutral buffered formalin for 30 min at room temperature and then incubated with hydrogen peroxide (Advanced Cell Diagnostics, San Francisco, CA, USA) for 10 min. Subsequently, samples were exposed to Protease plus (Advanced Cell Diagnostics, San Francisco, CA, USA) for 10 min before being incubated with specific probes diluted 1:50 in diluent buffer for 2 h at 40 °C in a HybEZ oven (Advanced Cell Diagnostics, San Francisco, CA, USA). The signal was amplified by incubating cells in signal amplification solutions 1, 2, and 3 and developed with the fluorophore OPAL 570 or OPAL 520 (Akoya biosciences, Marlborough, MA, USA), diluted 1:750 in tyramide signal amplification (TSA) buffer. Negative controls were performed by incubating cells with a probe specific for the *Bacillus subtilis* dihydrodipicolinate reductase (*dapB*) gene. Samples were counterstained with DAPI, and images were acquired using a fluorescent microscope (Nikon Eclipse TE200, Nikon, NY, USA). At least 7–8 images of 3 different wells (*n* = 3 replicates/well per each gene) of low and high seeding density for each cell line were acquired. Cells were considered positive for the target genes when containing one or multiple signal dots, as recommend by RNAscope technology guidelines. For all the genes, the percentage of cells positive for the specific target gene was calculated as the ratio of cells positive for the target genes on total cells counted for each scanned image. For *sox9*, gene expression intensity was also measured using the H-score, as recommended by the manufacturer. Briefly, expression was quantified using a five-level scoring system (0, no staining; 1, 1–3 dots/cell; 2, 4–10 dots/cell; 3, >10 dots/cell; 4, >15 dots/cell with > 10% of dots in clusters). The H-score was calculated as: (% of grade 1 cells × 1) + (% of grade 2 cells × 2) + (% of grade 3 cells × 3) + (% of grade 4 cells × 4).

### 2.7. Alkaline Phosphatase Activity

Determination of alkaline phosphatase was carried out using the Leukocyte Alkaline Phosphatase Kit (source, cat. n. 85L2-1KT), following the manufacturer’s instructions. Briefly, cells were fixed for 30 s at room temperature using a citrate/acetone solution. After fixation, alkaline phosphatase activity was revealed by incubation with the alkaline–dye mixture (Naphthol AS-MX and FAST BLUE RR salt solution) for 30 min at room temperature. Cells were then washed twice for 2 min using deionized water and nuclei were counterstained with hematoxylin. Brightfield images were acquired using a Nikon Eclipse TE200 microscope.

### 2.8. Statistical Analysis

Unless otherwise stated, results are presented as the mean ± standard deviation from at least three independent experiments. One-way ANOVA followed by Tukey’s post hoc tests were performed to perform multiple comparisons using PRISM version 8.2.1. (GraphPad Software, San Diego, CA, USA). Results were considered statistically significant when *p* < 0.05.

## 3. Result

### 3.1. Derivation of Novel Rainbow Trout Intestinal Cell Lines

**Figure 1:**
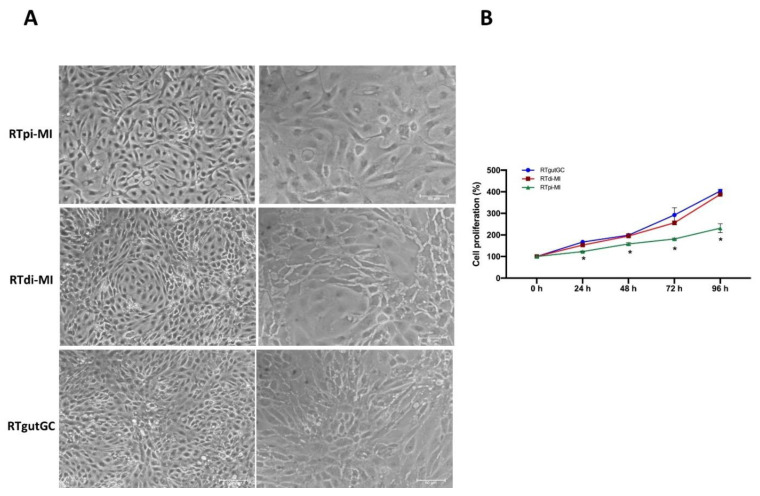
Morphology and cell proliferation of RTpi-MI, RTdi-MI and RTgutGC. (**A**) Representative picture of fully confluent RTpi-MI, RTdi-MI and RTgutGC cells at 20× (**left**) and 40× (**right**) magnification. Scale bar, 100 µm (left side) and 50 µm (right side). (**B**) Graphs reporting the growth curves of RTgutGC (blue line), RTdi-MI (red line) and RTpi-MI (green line) from 24 h (day 1) to 96 h (day 4) of culture showing that the RTpi-MI, derived from the proximal intestine, grew more slowly than RTgutGC and RTdi-MI cell lines, which were both derived from the distal intestine. Statistical significance for *p* < 0.05 is indicated as an asterisk (*). Data are reported as percentages (mean ± SD from 3 independent experiments) calculated on the cell seeding density for all cell lines.

Most fragments attached to the culture flasks and, after 5–10 days of culture, cells started to grow out from tissue explants but proliferated very slowly. In these early stages, cell morphology was heterogeneous and included fibroblast-like, dendritic-like, multipolar-like, and epithelial-like cells. After around 3 months of culture, fibroblast, dendritic and multipolar-like cells disappeared, probably entering a senescence state, leaving a quite uniform population of adherent epithelial-like cells. At this stage, tissue explants were removed, and outgrowths from each individual were detached enzymatically with a Trypsin-EDTA solution, pooled together, and plated in a single flask. Only two cell lines, one from the proximal and the other from the distal intestine of two different individuals attached and began to proliferate, reaching confluency within one week, whereas the cells from the remaining eight individuals did not. For routine maintenance, both RTdi-MI and RTpi-MI were plated in 25 cm^2^ flasks in L-15 medium with 5% FBS and were propagated at a 1:3 ratio after reaching the 90–95% confluence (Figure 1A). Both lines were able to resume vigorous growth after having been thawed following their storage in liquid nitrogen for up to 4 weeks.

The newly derived cell lines were named Rainbow Trout Proximal Intestine Milan Italy (RTpi-MI) and Rainbow Trout Distal Intestine Milan Italy (RTdi-MI) cell lines.

The RTpi-MI cell line displayed a slower proliferation rate (doubling time: 4.4 days) than the RTdi-MI (doubling time: 3.0 days) and RTgutGC (doubling time: 2.9 days) cell lines (Figure 1B).

### 3.2. Identification of Cell Types in RTpi-MI, RTdi-MI and RTgutGC Cell Lines

**Figure 2:**
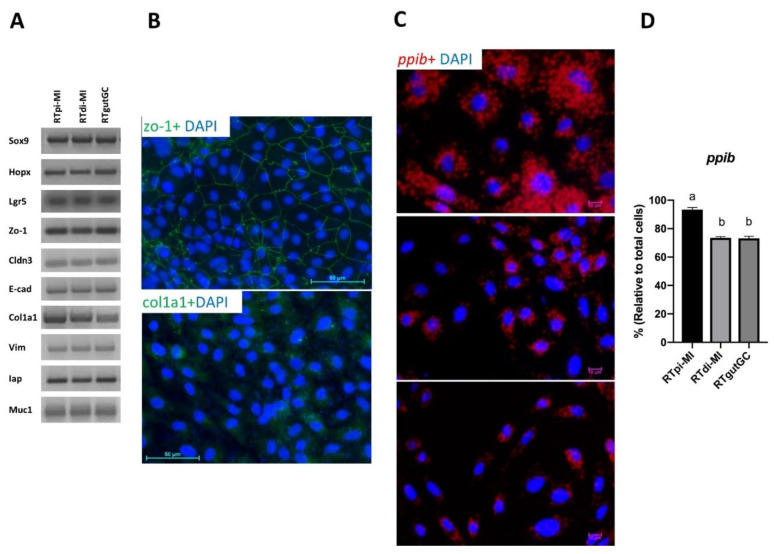
RTpi-MI, RTdi-MI and RTgutGC cell lines are composed of a heterogeneous population. (**A**) PCR amplicons of stem cell (*sox9*, *hopx* and *lgr5*), epithelial (*zo-1*, *cldn3* and *e-cad*) and connective tissue (*col1a1* and *vim*), enterocyte (*iap*) and goblet cell (*muc1*) genes in RTpi-MI, RTdi-MI and RTgutGC cell lines. (**B**) Representative immunofluorescence microphotographs showing zonula occludens (Zo-1, green, picture at the top) and collagen type 1 (Col1a1, green, picture at the bottom). DAPI (blue) was used to stain cell nuclei. Scale bar, 50 µm. (**C**) Representative immunofluorescence microphotographs showing the in situ hybridization of ppib (red) in RTpi-MI (picture at the top), RTdi-MI (picture in the middle) and RTgutGC (picture at the bottom). DAPI (blue) was used to stain cell nuclei. Scale bar, 10 µm. (**D**) Graphs reporting the percentage of *ppib*-expressing cells in RTpi-MI, RTdi-MI and RTgutGC cell lines. Data are reported as percentages (mean ± SD from 3 independent experiments) of *ppib^+^* cells on total cell count. Different superscript letters indicate statistical significance.

All cell lines expressed all the genes of the panel for cell type characterization (Figure 2A), indicating the presence of a heterogeneous population that included stem cells, immature differentiating cells, more differentiated epithelial cells expressing enterocyte and goblet cell markers, as well as connective cells. The presence of epithelial and connective tissue components was confirmed by positive staining of Zo-1 and Col1a1, respectively (Figure 2B). Interestingly, the percentage of mature and immature epithelial cells identified as expressing *ppib* by FISH was significantly higher in the RTpi-MI (92.8 ± 1.8%) cell line than in RTdi-MI (73.9 ± 0.7%) and RTgutGC (73.2 ± 1.5%; Figure 2C,D) cell lines.

### 3.3. Formation of a Functional Epithelial Barrier and Intracellular Absorption

**Figure 3:**
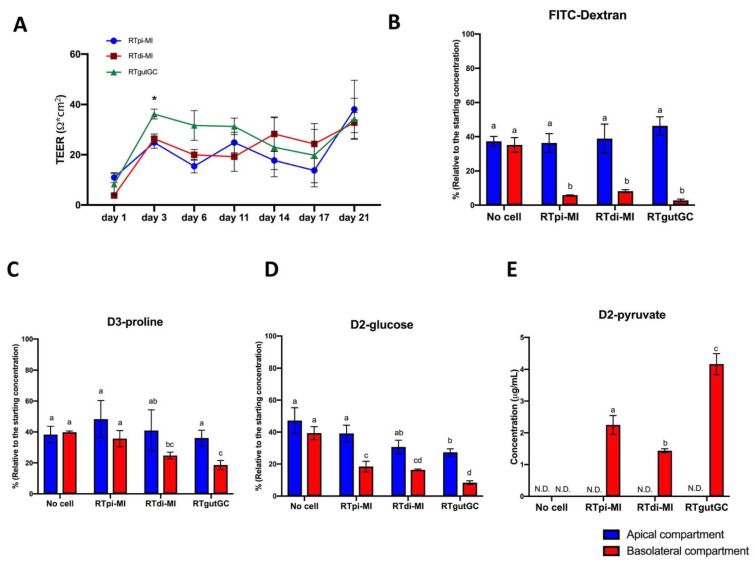
RTpi-MI, RTdi-MI and RTgutGC cell lines were able to constitute an intact and functional epithelial barrier. (**A**) Transepithelial electrical resistance (TEER) measured over 21 days of culture on a permeable PET membrane. Statistical significance for *p* < 0.05 is indicated as asterisk (*). (**B**) FITC-Dextran concentration (µg/mL) measured in the apical and basolateral compartments of the transwell after 24 h incubation. (**C**) D3-proline concentration (µg/mL) measured in the apical and basolateral compartments of the transwell after 24 h incubation. (**D**) D2-glucose concentration (µg/mL) measured in the apical and basolateral compartments of the transwell after 24 h incubation. (**E**) D2-pyruvate concentration (µg/mL) measured in the apical and basolateral compartments of the transwell after 24 h incubation without or with cells (N.D., not detected). Data in Figure (**A**) were obtained from 3 independent experiments run in parallel, whereas data in figures (**B**–**D**) were from 3 independent experiments. All data are shown as the mean ± SD. Different superscript letters indicate statistical significance.

Transepithelial electrical resistance (TEER) values, measured over 21 days of culture on PET membrane inserts, increased significantly from the baseline values already after 3 days (Figure 3A) and thereafter remined constant between 20 and 30 Ohm×cm^2^, indicating the formation of an effective barrier, with no significant differences among the three lines (Figure 3A). After 24 h incubation, the FITC-Dextran concentration was significantly higher in the apical than in the basolateral compartment in all cell lines, indicating the formation of a functional barrier that prevented the paracellular transport of large molecules (Figure 3B). Furthermore, all cell lines absorbed D3-proline and D2-glucose after 24 h incubation (Figure 3C,D). Interestingly, part of the glucose was metabolized into D2-pyruvate at different rates among the cell lines (Figure 3E).

### 3.4. Cell Plasticity

The effect of seeding density on cell line differentiation was evaluated by the quantification of stem cells (*sox9* and *lgr5*), differentiating cells (*hopx*), and differentiated enterocyte markers.

#### Cell Differentiation

**Figure 4:**
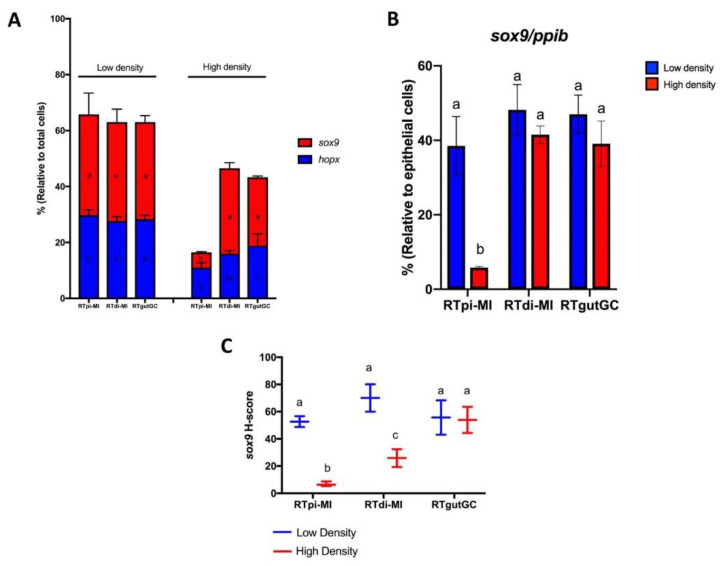

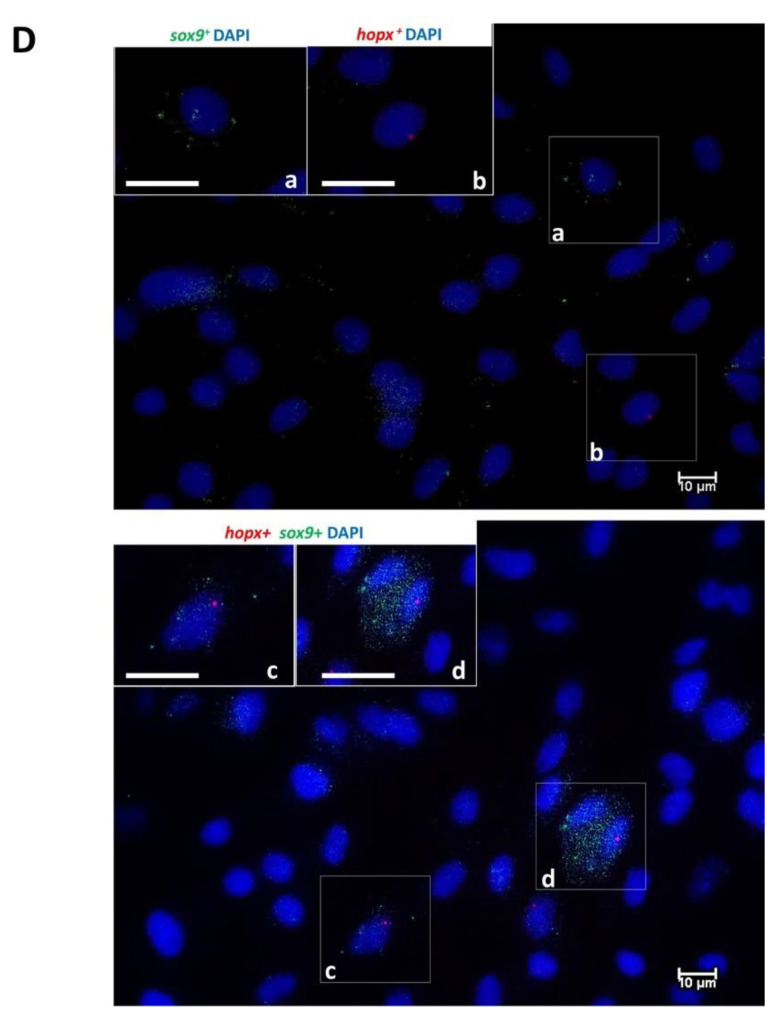
Effect of seeding density on *sox9* and *hopx* expression rate. (**A**) sox9^+^/hopx^*^ cell rates at low and high seeding densities relative to total cell count (top). Data are shown as the mean ± SD of 3 independent experiments. (**B**) *sox9+* cell rate at low (blue bars) and high (red bars) seeding densities relative to epithelial cells (*ppib^+^* cells, bottom). Data are shown as the mean ± SD of 3 independent experiments. (**C**) *sox9* h-score of RTpi-MI, RTdi-MI and RTgutGC at low and high seeding density. Data are shown as the mean ± SD of 3 independent experiments. Different superscript letters indicate statistical significance. (**D**) Representative microphotographs of cells only positive for either *sox9* or *hopx* (a,b) or both *sox9* and *hopx* (c,d). Scale bar, 10 µm.

Seven days after seeding at low density, *sox9*, a marker of intestinal stem cells in rainbow trout [13], was expressed in more than 30% of cells in all lines (Figure 4A). As expected, the proportion was higher when only epithelial cells, identified as expressing *ppib*, were considered (Figure 4B). In all cell lines, it was easy to detect variability in the signal intensity among positive cells (Figure 4A,B). Seeding at high density only inhibited overall *sox9* expression in RTpi-MI cells (Figure 4A,B). However, when expression intensity was measured using the H-score, it was possible to determine that also increasing the seeding density significantly inhibited *sox9* expression in RTdi-MI cells, whereas no effect was detected in the RTgutGC (Figure 4C).

At low seeding density, stem cells (*sox9*^+^) and differentiating (*hopx^+^*) cells made up more than 60% of the whole cell population in all cell lines but plating at higher density caused a significant downregulation of *hopx* expression, whereas only a few cells expressed both *sox9* and *hopx* at the same time (Figure 4D).

**Figure 5:**
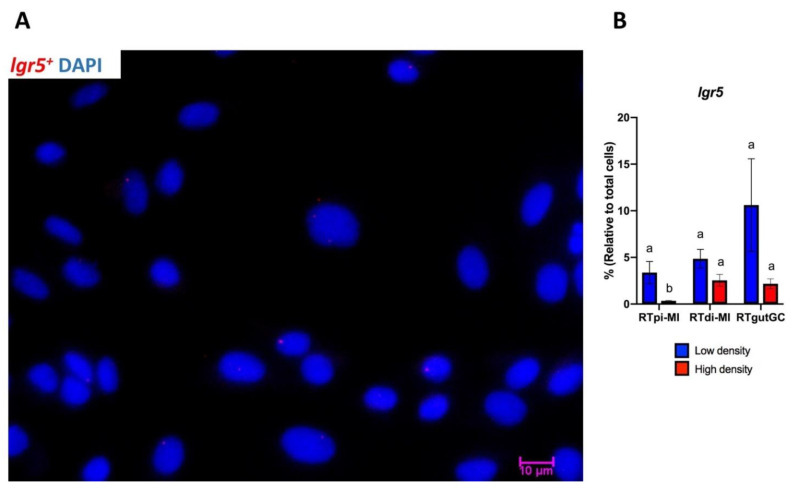
*Lgr5^+^* cell rates at low and high seeding density. (**A**) Representative microphotograph of *lgr5^+^* cells (red) at low and high seeding density. DAPI was used to stain the cell nucleus (blue). Scale bar, 10 µm. (**B**) *lgr5^+^* cell rates at low (blue bars) and high (red bars) seeding densities relative to total cell count. Data are shown as the mean ± SD of 3 independent experiments. Different superscript letters indicate statistical significance.

Finally, rare *lgr5^+^* cells were found in all cell lines (Figure 5A) and the effect of seeding density on this cell population was variable and inconsistent among the different lines, presumably due to their low number and relatively high variability (Figure 5B).

**Figure 6:**
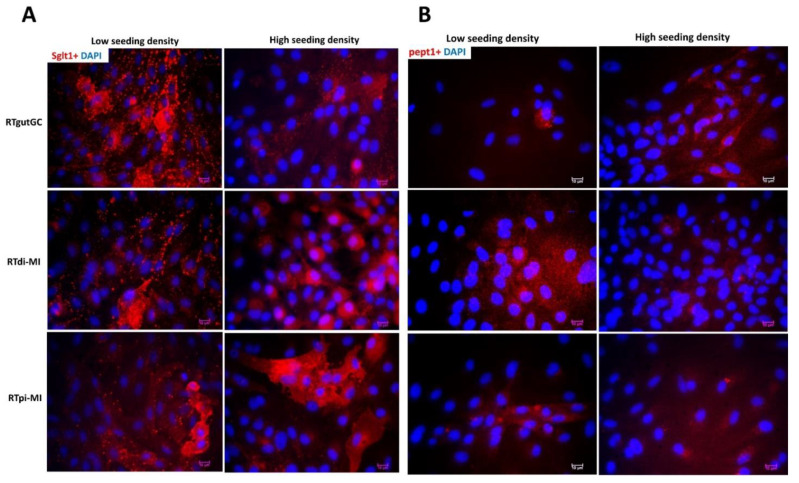
Protein expression of Sglt1 ((**A**), red) and Pept1 ((**B**), red) was not affected by cell seeding density. DAPI was used to stain cell nucleus. Scale bar, 10 µm.

In all cell lines, *sglt1* and *pept1* genes were expressed both at low and high seeding density (Figures S1 and S2, Supplementary Materials), whereas *fabp-2* expression was not detected (Figure S3, Supplementary Materials). The respective proteins were confirmed by immunofluorescence, showing specific Sglt1 and Pept1 signals (Figure 6A,B) and the absence of Fabp-2 (data not shown) in all cell lines, without an effect of cell seeding density. It is interesting to note that the Sglt1 signal was much stronger than PepT1 (Figure 6A,B).

**Figure 7:**
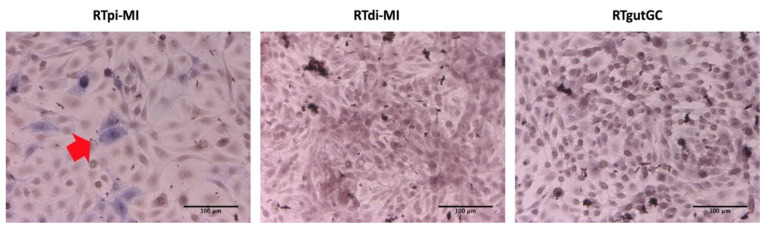
Alkaline phosphatase activity was only found in RTpi-MI at high seeding density (blue color, indicated by red arrows). Hematoxylin was used to stain the cell nucleus. Scale bar, 100 µm.

Alkaline phosphatase activity was used as evidence of functional enterocytes, and as shown, was influenced by seeding density. At low seeding density, enzyme activity was not detectable in any of the cell lines; instead, alkaline phosphate positive cells (blue) were only found in the RTpi-MI cell line seeded at high density (Figure 7).

## 4. Discussion

In this work, we derived two novel cell lines from the rainbow trout (RT) intestine, one from the proximal (RTpi-MI) and the other from the distal (RTdi-MI) intestine. These two cell lines were compared with the RTgutGC cell line, which was the only stable intestinal epithelial fish cell line available so far and was obtained from the distal intestine [17].

Although primary cell cultures from the different parts of the RT intestinal tract have been derived before, these cells had a finite lifespan of about 6 weeks [24]. Therefore, to the best of our knowledge, this makes RTpi-MI the first stable cell line derived from the proximal intestine of rainbow trout. At the time of writing, RTpi-MI and RTdi-MI have been successfully passaged more than 30 times, with no signs of senescence. They also survived four freezing and thawing cycles with no adverse effects on proliferation or behavior in culture (data not shown). These findings confirm the advantages of having stable cell lines for establishing in vitro models potentially available for a very long time. Indeed, the RTgutGC cell line, which was derived in a manner similar to that described here and has been passaged more than 100 times, constitutes a permanent resource of biological material that is being used in many research laboratories around the world.

On the other hand, in order to provide an in vitro model that can functionally replicate as many features as possible of the intestinal microenvironment, cell lines also need to preserve the heterogeneity that is characteristic of the intestinal epithelium in vivo. Indeed, this work represents the first to show that all cell lines include stem cells, differentiating and differentiated epithelial as well as connective cells.

Using in situ hybridization, we determined the proportion of epithelial cells expressing *ppib* in the cell population. This gene has been identified as a robust marker for identifying proliferating and differentiated mouse intestinal epithelial cells both in vivo and in vitro [25]. We also confirmed its role in rainbow trout showing that, in vivo, *ppib* was abundantly and exclusively expressed in every epithelial cell lining the mucosa of both the anterior and posterior intestine (Figure S4). Interestingly, epithelial cells were by far the largest but not the only component in all cell lines. However, *ppib* was significantly higher in the cell line derived from the proximal tract (RTpi-MI) than in the other two derived from the distal intestine (RTdi-MI and RTgutGC). At the same time, it was interesting to note that RTpi-MI cells had a lower proliferation rate than RTdi-MI and RtgutGC, a difference that mirrors the lower proliferation rate observed in vivo between the proximal and distal intestinal epithelium of the rainbow trout [20]. Overall, these findings indicate that these cell lines conserved at least some of the original complexity.

Consistent with their epithelial origin, RTgutGC were already known to express zonula occludens 1 [18,22,23,26,27], and we confirmed the expression of this tight junction protein in both RTdi-MI and RTpi-MI. We extended the epithelial characterization of all these lines, showing the expression of E-cadherin and Claudin, two molecules that interact with zonula occludens 1 for maintenance of the intestinal epithelium barrier and signal transduction between adjacent cells [28,29]. RTgutGC cells translate the expression of epithelial barrier genes into the formation of a functional barrier, as indicated by increasing TEER values measured at regular intervals up to 21 days after seeding [18,23,30,31,32]. Both RTpi-MI and RTdi-MI cell lines developed similar TEER values between 3 and 21 days of culture, which was close to the baseline resistance (50–400 Ω cm^2^) measured in in vivo studies [33,34]. These values are consistent with those of fish intestine and RTgutGC cells [23] that are considered as “leaky” epithelia [35]. Nevertheless, all cell lines formed a monolayer that significantly attenuated the permeation of FITC-Dextran 4000, a well-documented paracellular probe, widely used to study intestinal permeability in vitro [36]. This result confirms and extends previous observations describing RTgutGC as able to form a barrier that strongly attenuates dextran and albumin translocation from the apical to the basolateral chamber [18,27,32,37]. The functional selectivity of the barrier formed by the cell lines was confirmed by their capacity to absorb D2-glucose and D3-proline. Furthermore, the presence of deuterated pyruvate in the basolateral compartment after 24 h demonstrated that all cell lines metabolized D2-glucose upon absorption. These results clearly show that all cell lines were able to establish a polarized epithelium in vitro, in line with previous data [18,25,27]; in addition, this confirmed that glucose was not only absorbed but also metabolized, as previously shown in RTgutGC cells [22].

Vimentin and collagen type 1 are typically expressed by fibroblasts and myofibroblasts obtained from the human [31] and rainbow trout intestine [26], whereas the presence of a mixed population of epithelial and non-epithelial cells has never been explored before in intestinal cell lines of either fish or mammals. In vivo, the *lgr5*^+^ cells are located in the stromal tissue and are involved in the maintenance of tissue homeostasis by producing growth factors, cytokines, and proteins of the extracellular matrix [29]. In vitro, the presence of myofibroblasts enables the successful maintenance of human intestinal epithelial cell formation and proliferation beyond just a few days, even without the presence of supportive growth factors [38]. The ratio between myofibroblasts and epithelial cells remained constant over time, ranging approximately between 1–3 and 1–9, depending on the line, indicating the existence of a stable equilibrium between the two components. In vivo, *LGR5* expression in mice identifies the columnar basal cells that are considered the typical intestinal stem cells [39], but in rainbow trout, *lgr5^+^* cells are confined in the stroma [19]. However, recently, *LGR5*-expressing cells have been identified in the intestinal stroma also in mice [40], suggesting that these cells may play an important role in modulating epithelial renewal and differentiation in both species. Consistent with this hypothesis, we observed the presence of rare *lgr5*-expressing cells in all the lines, suggesting that their presence could be a possible cause of the ability of these RT cell lines to proliferate indefinitely or at least for a long time. Indeed, we obtained a cell line only from 2 out of 10 individuals, indicating that the success rate of this technique is relatively low, as also suggested by the fact that the current result represents the only successful replication so far of the derivation of stable rainbow trout intestinal cell lines, despite other systematic attempts [25]. Furthermore, the presence of both epithelial and stromal components is a desirable property for creating a physiologically relevant intestinal in vitro model [41].

As mentioned above, in mice and humans, *LGR5^+^* cells are the stem cells located at the bottom of the crypts which give rise to the proliferative progenitors that, in turn, will differentiate into all distinctive intestinal cell types [42,43]. However, recent data suggest that, in rainbow trout, this role is played by *sox9^+^* cells, whereas proliferative progenitors express *hopx,* and both cell types are found exclusively in the epithelium [19]. The stem cell and progenitor populations combined represent over 60% of all cells, a finding that is consistent with the self-renewal competence shown by all cell lines. Varying levels of *LGR5* expressions have been reported in 2D cultures of different human colorectal carcinoma cell lines, including the widely used Caco cells [44] in mouse primary colonic epithelial cell lines [45], and, at very low levels, in pig IPEC-J2 intestinal epithelial cell lines [46]. On the contrary, no data are available on the presence of progenitor cells in 2D culture conditions. The presence of very rare cells co-expressing *sox9* and *hopx* in the RT cell lines indicates that the two cell populations remain fully distinct in vitro, as observed in vivo, suggesting a high degree of maturation. In vivo, *sox9^+^* cells were found with different levels of signal intensity, and those with the highest intensity had the typical columnar base cell morphology that characterizes human and mouse bona fide intestinal stem cells [19]. Although the same morphology was not detectable *in vitro*, cells with different degrees of signal intensity were also observed in vitro, suggesting that at least a partial similarity was preserved even after several passages.

We used different seeding densities to test the ability of the cell lines to shift towards more differentiated cell phenotypes, as previously described in mammalian epithelial cell lines [47]. Indeed, the expression of all stem cell-related genes was downregulated by an increased seeding density, even if some differences among genes and cell lines were observed. *Hopx* expression was the most susceptible, being downregulated in all cell lines. This may be related to its expression by precursor cells that should be more prone to differentiation; therefore, increased cell density was enough to tip the balance in this direction. Similarly, the differentiation stimulus was more effective on cells expressing high levels of *sox9* that, in vivo, are suggested to be the bona fide equivalent of the typical mouse intestinal stem cells [19]. *Lgr5* expression, although detected in only few cells, was downregulated in RTpi-MI cells by high seeding density. *lgr5* should stimulate *sox9* and *hopx* expression while repressing cell differentiation; therefore, it appears as if a rudimentary stem cell niche is preserved in vitro.

However, current culture conditions can be improved because the downregulation of stem cell-related genes did not correspond to an equivalent upregulation of Sglt1 and PeptT1, either at the mRNA or protein level. This, together with the complete lack of expression of Fabp2, suggests that only a partial differentiation occurs in RTpi-MI following high seeding density. Nevertheless, the expression of *sglt1*, which actively transports glucose and galactose through the enterocyte’s apical membrane, was high and homogenous in all cell lines. More importantly, the Sglt1 transporter was fully functional, as indicated by the deuterated pyruvate measured in the basal compartment of the transwell PET membrane, a clear sign of glycolysis of absorbed glucose. Contrasting evidence was also obtained when alkaline phosphatase activity was measured. This enzyme has an important role in preserving gut mucosal defense [48,49] and has been used to identify terminally differentiated functional enterocytes [20]. In response to high seeding density, we found strong alkaline phosphatase activity, but only in the RTpi-MI cell line, consistent with its highest epithelial component and lowest cell proliferation rate, two parameters that, in vivo, favor cell differentiation. Previously, Kawano et al. 2011 [17] described the presence of alkaline phosphatase activity in very mature, high-density RTgutGC cultures. The apparent inconsistency could be related to the passage number or line-specific plating density requirements.

## 5. Conclusions

Two new stable cell lines, derived from the rainbow trout intestine, are now established and widen the choice of cell lines available for this species, including the proximal tract of the intestine. These cell lines provide an interesting addition to the rainbow trout invitrome [50], which indicates cell lines grouped around a common theme: in this case, the rainbow trout intestine. In particular, RTpi-MI is the first long-term cell line which has been established from the rainbow trout proximal intestine. Although most parameters were conserved among the three cell lines, some significant differences between the proximally and distally derived cell lines were observed, suggesting that some of the characteristics typical of each tract are conserved in vitro as well.

The presence of a mixed population of epithelial and stromal cells is an important aspect characterized for the first time and, in association with the expression of a regulatory molecule, such as Lgr5 by the stromal component, may explain why the cells can proliferate stably in vitro. At the same time, the lines include cells at different degree of differentiation and are able to respond to a mild differentiation stimulus, i.e., increased seeding density, with a downregulation of their stem cell component and increases in a few mature phenotypes. The use of culture procedures involving tridimensionality and physiological scaffold stiffness are possible ways to improve the control and the degree of differentiation that can be achieved.

## Supplementary Materials

The following are available online at https://www.mdpi.com/article/10.3390/cells10061555/s1. Table S1: List of primer sequences used for gene expression analysis. Gene ID, amplicon size in base pairs (bp), accession number ID, forward and reverse primer sequences are reported for each gene, Figure S1: Expression of sglt1 was found in RTgutGC, RTdi-MI and RTpi-MI at low (1–3 lane for each cell line) and high (4–6 lanes for each cell line) seeding density. A negative PCR control was run for assessing an absence of contamination, Figure S2: Expression of pept1 was detected in RTgutGC, RTdi-MI and RTpi-MI at low (1–3 lane for each cell line) and high (4–6 lanes for each cell line) seeding density. A negative PCR control was run for assessing at absence of contamination, Figure S3: No expression of fabp2 was found in RTgutGC, RTdi-MI and RTpi-MI at low (1–3 lane for each cell line) and high (4–6 lanes for each cell line) seeding density. A negative PCR control was run for assessing an absence of contamination, Figure S4: *ppib* mRNA was highly abundant in the epithelial cells of RT intestinal folds. Representative microphotographs showing *ppib* expression (red) in intestinal epithelial cells obtained by in situ hybridization. DAPI staining was used to identify the nucleus (blue). Scale bar, 100 µm, Supplementary Materials and Methods: Permeability test instrumentation operative conditions. References [30,32,51,52] are cited in the Supplementary Materials.
