# Supplementary material for: New Stable Cell Lines Derived from the Proximal and Distal Intestine of Rainbow Trout (Oncorhynchus mykiss) Retain Several Properties Observed In Vivo
Source: Cells. 2021 Jun 19;10(6):1555. doi: 10.3390/cells10061555 (PMC8235179; doi:10.3390/cells10061555)
Supplement: Supplementary file 1 [file cells-10-01555-s001.zip › cells-1245686-supplementary.pdf]

New stable cell lines derived from the proximal and distal intestine of rainbow trout (*Oncorhynchus mykiss*) retain several properties observed *in vivo*

Rolando Pasquariello[1], Nicole Verdile[1], Radmila Pavlovic[2], Sara Panseri[2], Kristin Schirmer[3], Tiziana A.L. Brevini[2], Fulvio Gandolfi[1]

Supplementary materials

Table S1. List of primer sequences used for gene expression analysis. Gene ID, amplicon size in base pairs (bp), accession number ID, forward and reverse primer sequences are reported for each gene.

Gene ID (amplicon size in base pairs (bp))	Accession Number ID	Forward (F) and reverse (R) Primer Sequence (5->3)	Annealing Temperature (°C)	Reference
sox9 (366bp)	NM_001124179.1	F:TGCAGGAGTGCATCTTTGTC<br>R:GGTCAGCCTTCTTGAACTCG	60	Own design
hopx (231bp)	XM_021584037.1	F:AGTAGAGCTTTCACACGGTCG<br>R:AGCTCTCAGCCACAGAAGTC	60	Own design
lgr5 (202bp)	XM_021576530.1	F:ACTCAACGGAGCCACAGATA<br>R:TTCTGGATCTTTCTGCAACCAC	60	Own design
zo-1 (99bp)	XM_021607172.1	F:GCTGTTCCTCCTAGACCTT<br>R:TCACCCACATCTGACTCTAC	60	[30]
cldn3 (112bp)	XM_021587920	F:AGGCAACGACGCTACATCAA<br>R:CCCTGGGAATCTACGTCAAA	60	[32]
e-cad (107bp)	XM_021607117	F:ACTATGACGAGGAGGGAGGT<br>R:TGGAGCGATGTCATTACGGA	60	[51]
col1a1a (214bp)	NM_001124207	F:CTTTGCTGCTCAGTTTGATGG<br>R:GTTACCATCCTCACCAGGTTT	60	Own design
vim (203bp)	NM_001124729.1	F:AGGAAGGTGGAGTCTCTTCA<br>R: TCTTCAGAGTCCTGGAGGTT	60	Own design
iap (157bp)	XM_021606337	F:TTGGCACGGAAAGAGAGTCC<br>R:CATGGGAAAGCTACCAGGCA	60	[32]
pept-1 (161bp)	XM_031818746.1	F: CCTGTCAATCAACGCTGGT<br>R: CACTGCCCATAATGAACACG	60	[52]
sglt1 (208bp)	XM_021591066.2	F:ATCGCTGAGTTTGCCTATGG<br>R:TCTCCTCTGTGTGGTTCCT	60	Own design
fabp2 (201bp)	XM_021619074.1	F:GACAACTACCAGAGCCATTGT<br>R:AAAGACCTAAAGTGGTCCAGTG	60	Own design
muc1 (263bp)	XM_021578564.1	F:GTCTGCCACATGCCAAATATC<br>R:CCCTCCATGAATGAGTCACATA	60	Own design

References:

30. Schug H, Yue Y, Krese R, Fischer S, Kortner TM, Schirmer K. Time- and concentration-dependent expression of immune and barrier genes in the RTgutGC fish intestinal model following immune stimulation. Fish Shellfish Immunol. 2019 May;88:308-317. doi: 10.1016/j.fsi.2019.02.036.

32. Wang, J.; Lei, P.; Gamil, A.A.A.; Lagos, L.; Yue, Y.; Schirmer, K.; Mydland, L.T.; Øverland, M.; Krogdahl, Å.; Kortner, T.M. Rainbow trout (Oncorhynchus mykiss) intestinal epithelial cells as a model for studying gut immune function and effects of functional feed ingredients. Front. Immunol. 2019, 10, doi:10.3389/fimmu.2019.00152.

51. Hu H, Kortner TM, Gajardo K, Chikwati E, Tinsley J, Krogdahl Å. Intestinal Fluid Permeability in Atlantic Salmon (Salmo salar L.) Is Affected by Dietary Protein Source. PLoS One. 2016 Dec 1;11(12):e0167515. doi: 10.1371/journal.pone.0167515.

52. Ostaszewska T, Kamaszewski M, Grochowski P, Dabrowski K, Verri T, Aksakal E, Szatkowska I, Nowak Z, Dobosz S. The effect of peptide absorption on PepT1 gene expression and digestive system hormones in rainbow trout (Oncorhynchus mykiss). Comp Biochem Physiol A Mol Integr Physiol. 2010 Jan;155(1):107-14. doi: 10.1016/j.cbpa.2009.10.017. Epub 2009 Oct 22. Erratum in: Comp Biochem Physiol A Mol Integr Physiol. 2010 Aug;156(4):569.

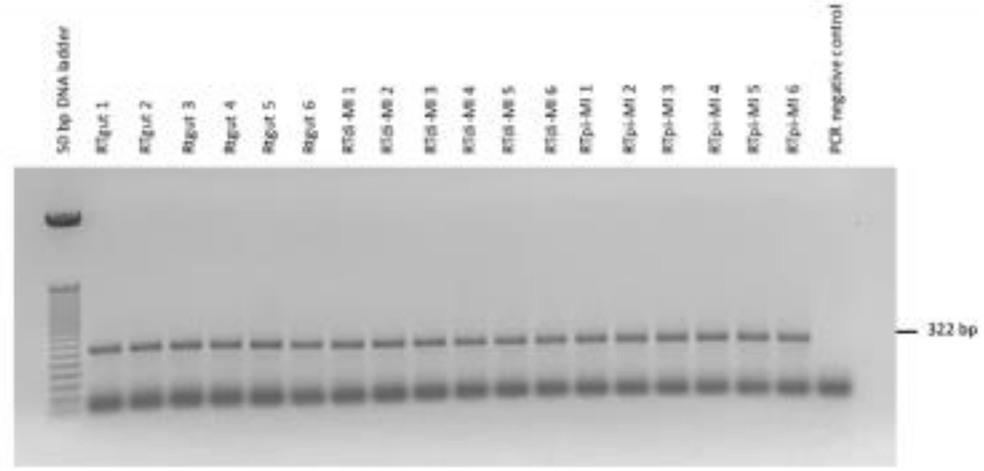

Figure S1. Expression of *sglt1* was found in RTgutGC, RTdi-MI and RTpi-MI at low (1-3 lane for each cell line) and high (4-6 lanes for each cell line) seeding density. A PCR negative control was run for assessing at absence of contamination.

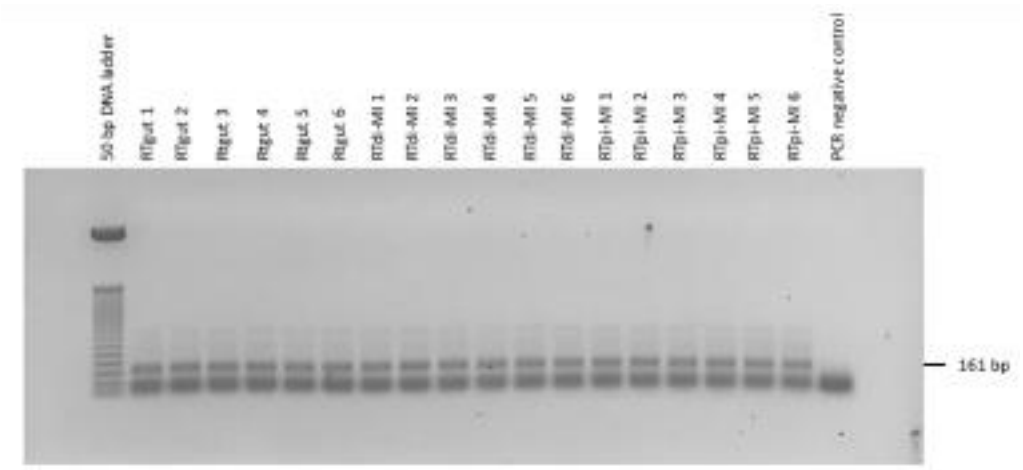

Figure S2. Expression of *pept1* was detected in RTgutGC, RTdi-MI and RTpi-MI at low (1-3 lane for each cell line) and high (4-6 lanes for each cell line) seeding density. A PCR negative control was run for assessing at absence of contamination.

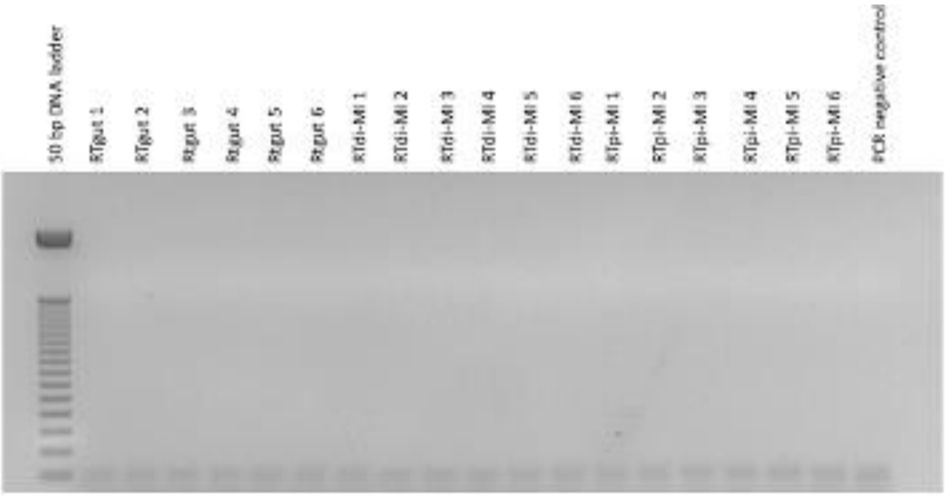

Figure S3. No expression of *fabp2* was found in RTgutGC, RTdi-MI and RTpi-MI at low (1-3 lane for each cell line) and high (4-6 lanes for each cell line) seeding density. A PCR negative control was run for assessing at absence of contamination.

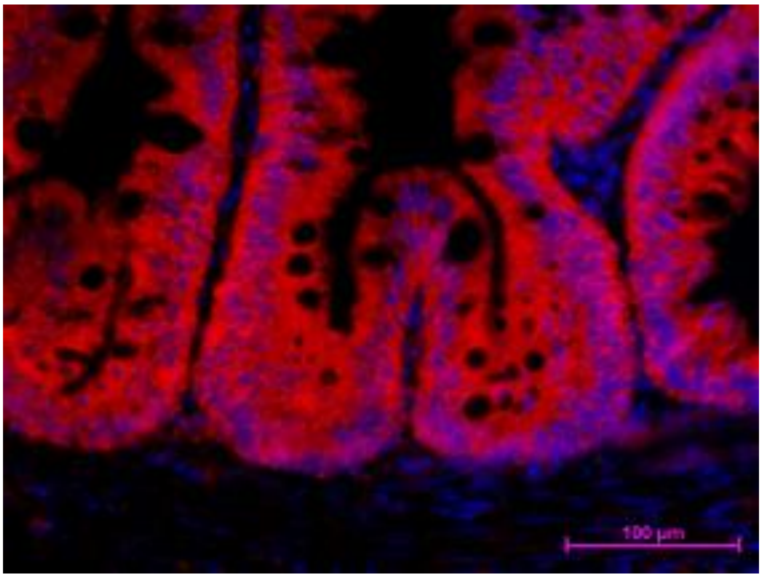

Figure S4. *ppib* mRNA was highly abundant in epithelial cells of RT intestinal folds. Representative microphotographs showing ppib expression (red) in intestinal epithelial cells obtained by in situ hybridazation. DAPI staining was used to identify nucleus (blue). Scale bar, 100 µm.

Supplementary materials and methods:

Permeability test Instrumentation operative conditions

Glucose-D2 and Proline-D3 determination

High performance liquid chromatography (Vanquish UHPLC) system coupled to high resolution mass spectrometer q-Exactive Orbitrap (HPLC-Orbitrap-HRMS) (Thermo Fisher Scientific, San Jose, CA, USA) was used for Glc-D2 and Pro-D3 determination. Operative conditions were as follows:

Synergy Hydro RP column (150 × 2 mm i.d., 4 µm, (4×3 mm i.d.) (Phenomenex, Torrance, CA, USA) was used as stationary phase. The mobile phase involved water acidified with 0.1% formic acid (A) and methanol (B). The gradient (0.3 mL/min flow rate), begun with 98% of eluent A with a linear reduction to 50% in 5 min, that was constant in the next 3min. The initial conditions returned at 8 min, tracked by a 5-min re-equilibration period. The column department temperature was set at 30°C whereas autosampler was kept at 5°C.

Q-Exactive Plus HRMS operated in both positive and negative mode with the following operative parameters of heated electrospray ionisation (HESI) source: 3.4 kV for capillary voltage, 290°C for capillary temperature and 280°C for auxiliary gas heater temperature. Sheath and auxiliary gas were adjusted at 35 and 15 arbitrary units, while S lens RF level was set at 55. Resolving power of full scan (FS) adjusted on 70,000 FWHM at m/z 200, with scan range of *m/z* 80-500. The automatic gain control (AGC) was set at $3e^6$, with an injection time of 100 ms. Detection was based on calculated exact mass of the protonated/deprotonated molecular ions, at least one corresponding fragment and on retention time of target compounds. Extracted ion chromatograms (EICs) were obtained with an accuracy of 2 ppm *m/z* from total ion chromatogram (TIC) engaging the *m/z* corresponding to the molecular ions: $[M-H]^-$=181.06866 for Glc-D2 and $[M+H]^+$= 119.08944 for Pro-D3. As an example, the EIC of one real sample is presented below (**Figure S5**).

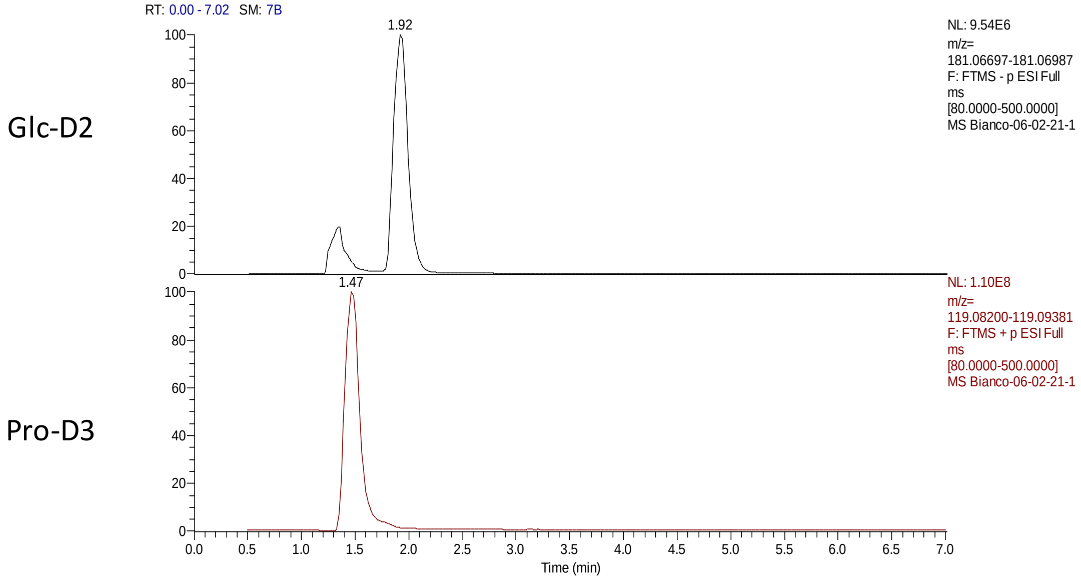

Figure S5. EICs of Glucose-D2 and Proline-D3 in initial L-15 medium

FITC-dextran determination

High performance liquid chromatography system with fluorometric detector (Jasco) was employed for FITC-Dex quantification. Thermo Scientific™ Hypersil ODS C18 HPLC (50 × 2 mm i.d., 5 µm) served as a stationary phase while mobile one consisted of water (A) and methanol (B). The gradient (0.75 mL/min flow rate), begun with 60% of eluent A with a linear reduction to 10% in 5 min, that was constant in the next 4min. The initial conditions returned at 9 min, followed by 5-min re-equilibration period. The column department temperature was set at 30°C and while autosampler was kept at 4°C. The excitation and emission wavelengths were 490 and 520 nm, respectively, for FITC-dex. As an example, three overlapping chromatograms (initial L-15 medium, upper and basolateral compartments of the same sample is presented below (**Figure S6).**

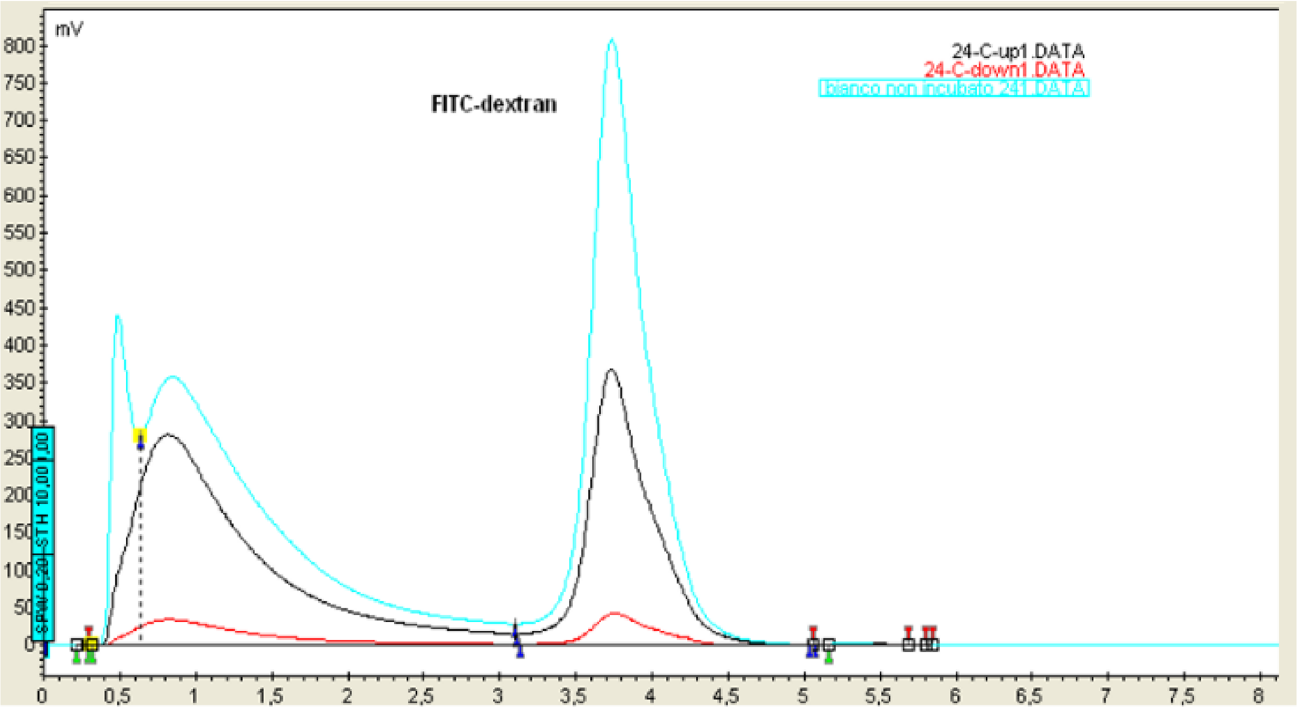

Figure S6. FITC-Dex in the initial medium (blue), upper (black) and basolateral (red) compartments
